# γδ T Lymphocytes as a First Line of Immune Defense: Old and New Ways of Antigen Recognition and Implications for Cancer Immunotherapy

**DOI:** 10.3389/fimmu.2014.00575

**Published:** 2014-11-11

**Authors:** Alessandro Poggi, Maria Raffaella Zocchi

**Affiliations:** ^1^Unit of Molecular Oncology and Angiogenesis, IRCCS-AOU San Martino-IST, Genoa, Italy; ^2^Division of Immunology, Transplants and Infectious Diseases, IRCCS San Raffaele, Milan, Italy

**Keywords:** γδ T cells, aminobisphosphonate, BTN3A1, NKG2D ligands, ADAM proteins

## Abstract

Among γδT cells, the Vδ1 subset, resident in epithelial tissues, is implied in the defense against viruses, fungi, and certain hematological malignancies, while the circulating Vδ2 subpopulation mainly respond to mycobacteria and solid tumors. Both subsets can be activated by stress-induced molecules (MIC-A, MIC-B, ULBPs) to produce pro-inflammatory cytokines and lytic enzymes and destroy bacteria or damaged cells. γδT lymphocytes can also recognize lipids, as those associated to *M. tuberculosis*, presented by the CD1 molecule, or phosphoantigens (P-Ag), either autologous, which accumulates in virus-infected cells, or microbial produced by prokaryotes and parasites. In cancer cells, P-Ag accumulate due to alterations in the mevalonate pathway; recently, butyrophilin 3A1 has been shown to be the presenting molecule for P-Ag. Of interest, aminobisphosphonates indirectly activate Vδ2 T cells inducing the accumulation of P-Ag. Based on these data, γδT lymphocytes are attractive effectors for cancer immunotherapy. However, the results obtained in clinical trials so far have been disappointing: this review will focus on the possible reasons of this failure as well as on suggestions for implementation of the therapeutic strategies.

## γδ T Cells and Antigen Recognition

Human γδ T lymphocytes comprise different subsets defined by their T-cell receptor (TCR), the most prominent of which is present in circulating blood, representing 3–5% of T lymphocytes, and is composed of cells expressing the Vγ9Vδ2 TCR (Vδ2T cells). The subset bearing the Vδ1 chain of the TCR is <1–2% of circulating T cells and is mostly represented in the mucosal-associated lymphoid tissue, known to play an important role in the first-line defense against viral, bacterial, and fungal pathogens (1–5). γδT cells recognize a wide variety of antigens, such as lipids, proteins, and phosphoantigens (P-Ag), without the need of HLA-restricted antigen presentation (6–9): circulating Vδ2 T lymphocytes are involved in the response to mycobacteria, EBV, and some solid tumors, while resident Vδ1 T cells contribute to the immunity against *Listeria monocytogenes*, CMV, and certain hematological malignancies (2–4, 10). Both γδT-cell subsets can interact with stress-induced MIC-A, MIC-B, and ULBPs; the recognition is mediated through the NKG2D receptor, also expressed by αβT lymphocytes (3, 11–13). In γδT cells, NKG2D seems to work in association with the TCR that also binds to these stress molecules: upon its engagement, an activating signal is delivered in γδT lymphocytes that promptly exert their effector function, by proliferating, producing pro-inflammatory and antimicrobial cytokines, such as interferon-gamma (IFN)-γ or tumor necrosis factor (TNF)-α, or releasing lytic enzymes to destroy bacteria or infected cells, as a response to damage signals (10–13). A similar mechanism can be exploited by γδT lymphocytes to face transformed cells that also overexpress NKG2D ligands (−L) due to the stress-inducing transformation, like in solid tumors or in hematological malignancies (14–19). Of note, these ligands can also be upregulated at the cell surface by drugs, including all-trans-retinoic acid or sodium valproate, commonly used in anti-leukemic therapeutic schemes, thus improving γδ T cell-mediated anti-cancer capacity (20–23). Another potent stimulus for γδT cells of the Vδ2 subset, acting through the TCR, is represented by low molecular weight P-Ag (4–8). Consistent with the stress-surveillance model, P-Ag may be autologous, such as isopentenylpyrophosphate (IPP), which accumulates in many virus-infected or transformed cells, or microbial, such as hydroxymethyl but-2-enyl pyrophosphate (HMBPP), a metabolic intermediate specific to many prokaryotes and parasites (4–8). Of clinical interest, aminobisphosphonates (N-BPs), which are widely prescribed for osteoporosis and malignancy, indirectly activate Vγ9Vδ2 cells by inhibiting farnesyl-pyrophosphate synthase, which provokes IPP accumulation (24–28).

## Possible Antigen-Presenting Molecules for γδ T Cells

Thus, the types of Ag recognized by γδT lymphocytes may vary in size, composition, and molecular structure, much more than those recognized by αβT cells, and include soluble or cell surface proteins, small peptides, phospholipids, prenyl-pyrophosphates, and sulfatides. The mode of antigen recognition by γδT cells has been a controversial issue for several years, as they apparently do not need Ag presentation by specialized cells. The TCR that these lymphocytes are equipped with, display some peculiar features such as a limited diversity compared to the αβTCR, and a type of interaction with the Ag that rather resembles that of the B-cell receptor. This hypothesis is based on structural and functional findings: indeed, CDR3 regions of the γδTCR resemble immunoglobulin (Ig) CDRs in terms of length and variability, as the TCRδ and γ chain have long or short CDR3, respectively, as is the case of Ig heavy and light chains (29, 30). In contrast, length and conformation of TCRα and β CDR3s are similar to each other, which may be a requirement for the docking on the surface of MHC molecules and the recognition of MHC-bound peptides. In some cases, however, small Ag may be presented to γδT cells as well, in general in the case of soluble small molecules unable to induce a TCR cross-linking (31). A still unsolved question seems to be the Ag-presenting molecule recognized by γδT cells. In mice, the non-classical or truncated MHC molecules T10/T22, not constitutively expressed at the cell surface but induced by stress signals, have been shown to bind to γδTCR, that makes an angle using CDR3δ amino-acid side chains for the interaction (32, 33).

Other structures described to be potentially responsible for Ag presentation to γδT cells are the group1 CD1 molecules. CD1 comprises a family of non-polymorphic genes located outside the MHC complex and encodes proteins structurally related to MHC class-I molecules (34, 35). In humans, products of four of the five CD1 genes, designated CD1a, CD1b, CD1c, and CD1d, have been identified as type 1 integral membrane proteins associated with β2-microglobulin and are expressed on antigen-presenting cells. A direct evidence for CD1 proteins as antigen-presenting molecules was provided by isolation of a human CD4^−^CD8^−^ T-cell line that proliferated in response to *M. tuberculosis*-derived antigens: the purification of the CD1b-restricted antigens revealed a subset of mycolic acids, a family of free fatty acids present in the outer cell wall of mycobacteria and several other bacteria. Soon after, some glycolipids, such as phosphatidylinositol-containing lipoglycans and glycosylated mycolates, that are also associated with the mycobacteria cell wall, were identified as CD1b-presented antigen (36, 37). The CD1-restricted presentation of lipid and glycolipid antigens to T cells was strengthened by the three-dimensional structure of the mouse CD1d protein determined by X-ray crystallography (35, 38), showing a putative antigen-binding groove, which is remarkably different from that found in MHC molecules. Subsequent characterization of mycobacteria-derived antigens revealed a remarkable ability of human group 1 CD1 (CD1a, CD1b, CD1c) to mediate presentation of lipid and glycolipid antigens to T cells, including γδT cells.

It has been unknown for many years whether and how prenyl-pyrophosphates are presented to γδT cells. In the last two years, a number of papers have been published identifying butyrophilin (BTN)3A1 as the molecule that can directly bind P-Ag for presentation. BTNs are type 1 trans-membrane molecules containing two Ig-like domains in their extracellular portion (39). Some BTNs carry a B30.2 domain. In humans, the BTNs genes are clustered on chromosome 6 in the MHC class-I region containing three related genes: BTN3A1, BTN3A2, and BTN3A3 (40, 41). The former molecule seems the only one containing a B30.2 domain, forming a basic pocket, which is essential for N-BPs-mediated activation of γδT cells, although the authors did not show evidence for direct binding of P-Ag to BTN3A1 (40). More recently, such direct binding has been demonstrated to occur to the V-like domain of BTN3A1 and the complex has been crystallized (42). It is still not clear how intracellularly generated P-Ag (e.g., those derived upon N-BPs treatment) can be associated to BTN3A1: one possibility is that P-Ag are secreted and then bind to the basic groove of BT3A1 or, alternatively, the B30.1 basic domain binds to P-Ag with low affinity and induces a conformational change in the external portion of the molecule that, in turn, is recognized by γδ T cells (39, 42, 43).

## γδ T Cells and Anti-Cancer Surveillance

Since their discovery in the late 1980s, γδT cells have been extensively studied and different characteristics, including MHC-unrestricted cytotoxic activity against malignant cells, have made these cells a promising potential therapeutic tool (3, 4, 10, 15, 44–46). It is now clear that γδT lymphocytes are good mediators of a stress-related response: for example, they can recognize directly stress-induced ligands, such as MIC-A, MIC-B, or ULBPs, through the NKG2D receptor or be activated by P-Ag derived by the isoprenoid pathway used by several microorganisms or by the mevalonate pathway in infected or transformed cells (1–4). However, NKG2D-L can be released, due to the action of the disintegrin-and-metalloproteinases ADAM10/17 or the disulfide-isomerase ERp5, overexpressed in solid and hematologic tumors (47–52). In their soluble form (sNKG2D-L), these ligands hinder the recognition of membrane-bound MIC-A/B or ULBPs by NKG2D receptor; in turn, sNKG2D-L are not able to trigger an activating signal in effector lymphocytes that cannot exert their anti-tumor activity (46–51). Moreover, serum levels of sNKG2D-L have been related to the outcome and progression of several neoplastic diseases (18, 23, 52–54).

γδT cells can also be indirectly activated by pro-inflammatory cytokines or by toll-like receptors (TLR) that bind to viral or bacterial products (1–4). Another activation signal can be delivered via CD16 through the interaction with the Fc of IgG: this binding initiate the antibody-dependent cell cytotoxicity (ADCC) exerted to destroy opsonized cells or microorganisms (2). Upon one of the mentioned stimuli, γδT lymphocytes expand, acquire cytotoxic function, and secrete an array of Th1 pro-inflammatory cytokines, such as IFN-γ or TNF-α. Another important feature of T lymphocytes expected to interact with cancer cells is their capacity to infiltrate tumors. Accordingly, tumor-infiltrating gamma delta T lymphocytes were detected in a broad spectrum of malignancies (2–4, 10).

For all these aspects of their function, γδ T cells have been considered attractive for anti-cancer therapies: of note, ADCC can be exploited by the use of therapeutic monoclonal antibodies (mAbs) (44, 45, 55). In addition, various selective agonists, including P-Ag, for human γδT lymphocytes have been synthesized, allowing the launch of several clinical trials for patients with follicular lymphoma, multiple myeloma (MM), and acute myeloid leukemia, as well as non-hematological malignancies, such as renal cell (RCC), breast, and prostate carcinomas.

## Evaluation of γδ T Cell-Based Clinical Trials

Given the demonstrated *in vitro* anti-cancer activity of γδT cells and their *in vivo* potential as anti-tumor effectors, numerous clinical trials have been performed in the last years to exploit the properties of these cells for cancer immunotherapy (44, 56–64). Two methods have been applied so far: adoptive transfer of autologous γδT lymphocytes expanded *in vitro* and then reinfused to patients and direct administration of drugs or substances able to stimulate γδT cells *in vivo* (44, 56–58). The *in vitro* stimulation and expansion of this cell population is achievable using P-Ag, N-BPs, or immobilized anti-γδ TCR antibodies, and allows the optimization and control of the effector cells obtained (7, 8, 24, 56). However, this method requires specialized laboratories and expertise and is rather expensive. In turn, the administration of N-BPs or synthetic P-Ag in combination with cytokines has been used as a cheaper and straight-forward therapeutic alternative. The third generation of N-BPs as zoledronate is the most commonly used for both *in vitro* activation and *in vivo* administration; the EC_50_ for γδ T cells is favorable (0.003 μM) and a single dose of 4 mg leads to plasma levels (1–5 μM) shown to be effective in activating γδT cells *in vitro* (56, 60). As an alternative, the synthetic phosphate-containing molecule bromohydrin pyrophosphate (BrHPP) is used for either *in vitro* expansion or *in vivo* stimulation of γδ T lymphocytes and also upregulates their ability to mediate rituximab-induced ADCC (56, 61). Together with zoledronate or BrHPP, interleukin-2 is used for *in vitro* expansion of this T-cell population, and also added to the therapeutic schemes in different cancers; however, IL-2 is toxic at high doses (those that are commonly effective), leading to vascular leakage, hyperpyrexia, severe hypotension whereas low, and well-tolerated doses are much less effective *in vivo* (28, 56).

A preliminary pilot study by Wilhelm’s team examined toxicity, *in vivo* activation of γδT cells, and anti-lymphoma efficacy of pamidronate/IL-2 in 19 patients with relapsed/refractory low-grade non-Hodgkin lymphomas (NHL) or MM (44). The authors demonstrated that pamidronate administered with low-dose IL-2 is well tolerated and induces a specific γδT-cell expansion; furthermore, the clinical response observed in the patients, i.e., stabilization or partial response, is linked to γδT-cell proliferation *in vivo*. A second study was reported by Dieli’s group, showing that zoledronate induced the *in vivo* development of Vγ9Vδ2 cells producing IFN-γ and exerting strong anti-tumor responses (62). Therefore, a pilot study on the effects of zoledronate and IL-2 was conducted in the United States by Malkovsky’s group in 12 patients with metastatic RCC (63). Adverse events typical of IL-2 monotherapy were observed in all patients, without partial or complete responses. In the following years, phase-I clinical trials were performed in metastatic hormone-refractory prostate cancer and in several patients with solid tumors using BrHPP (56, 64). Given BrHPP’s safety profile, a multicentric phase-II study using the drug was launched in relapsed follicular lymphoma patients who had previously received previous lines of therapy, using rituximab at least once (56, 61). The treatment induced strong and specific amplification of TCRVγ9Vδ2 T lymphocytes showing a Th1 and cytotoxic effector-memory cell profile (IFN-γ and TNF-α production), expressing FcγRIIIa (CD16) and displaying rituximab-mediated ADCC (56, 61). The combination of BrHPP and rituximab in immunotargeted therapy produced very encouraging results, particularly for follicular lymphoma patients with unfavorable FcγRIIIa gene polymorphisms (F/F or V/F, 95% of the patients). Thus, the initial evaluation of clinical trials leads to the conclusion that γδT cell-based immunotherapy is more effective in hematological rather than in solid tumors.

## Possible Improvement of γδT Cell-Based Immunotherapy

In the above cited review by Fisher and coworkers (56), 12 clinical trials involving 157 patients have been analyzed for the evaluation of the efficacy and/or failure of γδT cell-based immunotherapy, and some conclusions can be drawn. First, patients with solid tumors have been treated mostly with adoptive γδ T-cell transfer, while patients with hematological cancers were mainly treated with γδ T cell-expanding drugs. Second, as the trials reviewed were either phase-I, phase-II, or feasibility studies, all patients had already received previous treatments, as chemotherapy or other types of immunotherapy (IL-2 alone). Moreover, in some trials testing γδT cell-stimulating drugs, the combination with IL-2 led to high toxicity with low therapeutic effects. In adoptive transfer studies, different culture conditions and times as well as distinct cell sources (leukapheresis vs. peripheral blood), represent additional variables that render difficult the overall evaluation of the efficacy of these treatments. As the *in vitro* expansion of γδT lymphocytes is feasible and efficient, an accepted conclusion is that leukapheresis in general is not needed to obtain a sufficient amount of activated effectors to reinfuse. Some evidences emerge from the comparison of clinical responses to γδT cell-immunotherapy with standard-of-care second-line therapies in three selected cancer types, RCC, non-small cell lung carcinoma (NSCLC), and prostate cancer. The proportion of objective responses among patients treated with γδT cell-based immunotherapy is higher than that achieved with recommended second-line therapy in advanced prostate cancer (33.3% with γδT cells vs. 25.2% with prednisolone + docetaxel) and advanced RCC (4.8% with γδT cells vs. 1.8 with everolimus), but not in advanced NSCLC (7.6% with erlotinib, 12.2% with docetaxel, 0% with γδ T cells) (56, 65–67). In general, the clinical response to γδT-cell immunotherapy in solid tumors is disappointing. There are several possible explanations for this and we will try to consider some of them. First, there might be a considerable difference in γδT-cell expansion capacity among patients, patients with hematologic malignancies being more responsive that those with solid tumors (44, 56–64). A considerable inter-individual variation in expansion capacity has been observed among patients with MM, NHL, or chronic lymphocytic leukemia (CLL) with an inverse correlation between the frequency of circulating regulatory T cells and the ability of γδT cells from cancer patients to proliferate in response to P-Ag (44, 56–64). Another possible inhibiting factor is represented by transforming growth factor (TGF)β that is known to decrease the NKG2D expression on lymphocytes reducing their activation (52, 68, 69). Moreover, sNKG2D-L released by cancer and accessory cells in the tumor microenvironment can impede the interaction of effector lymphocytes with tumor target cells. (48–52) In addition, other inhibiting signals, such as that delivered by PD-1 or via CTLA-4, can lead to a general inhibition of γδT-cell function at the tumor site (70, 71). Thus, a possible strategy to overcome inhibitory signals would be the use of mAbs blocking either CTLA-4, such as ipilimumab, or PD-1 or neutralizing TGFβ (56, 69–71). In addition, inhibiting the enzymes responsible for sNKG2D ligands, including ADAM10 and ADAM17 (71–76), with specific compounds, would push the balance toward γδT-cell activation; along this line, the combination of stimulating molecules, such as bisphosphonates, and therapeutic tumor-targeting antibodies, as the anti-CD20 rituximab or the anti-ERBB2 trastuzumab, should improve the efficacy of γδT-cell anti-tumor effect (56, 58). A different immunoevasion mechanism exerted by tumor microenvironment may be represented by mesenchymal stromal cells (MSC) that are known to down-regulate T-cell effector functions (77, 78). We recently reported that LN-MSC derived from NHL patients impair the anti-tumor activity of Vδ2T lymphocytes, selectively inhibiting NKG2D-mediated lymphoma cell killing (79). Of note, N-BPs can prevent this effect by reducing TGFβ and increasing IL-15 production by LN-MSC, and drive the differentiation of Vδ2 T lymphocytes into effector-memory cells producing Th1-type cytokines (79). Moreover, N-BPs do not alter the efficiency of Vδ2 T cells to exert rituximab-mediated ADCC. To be successful, γδT cell-based cancer immunotherapy will require protocols updated to limit most of the different immunoescape mechanisms occurring at the tumor site.

## Perspectives

**Figure 1:**
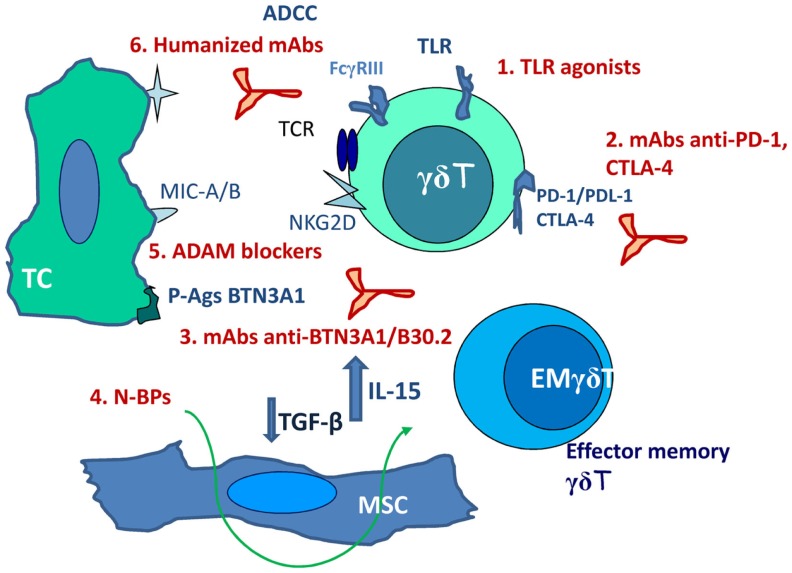
**Scheme of possible combinations of activating and inhibiting stimuli aimed to potentiate γδT-cell anti-cancer response**. 1. TLR agonists (imiquimod or resiquimod). 2. mAbs blocking PD-1, PDL-1, and CTLA-4. 3. mAbs directed to the B30.2 basic domain of BTN3A1. 4. N-BPs not only as γδ T-cell stimulating agents but as immunomodulating drugs (decrease TGF-β and increase IL-15 production by LN-MSC). 5. ADAMs specific and non-toxic inhibitors. 6. Humanized mAbs directed against tumor antigens (rituximab, trastuzumab). TC, tumor cell; EM, effector memory; MSC, mesenchymal stromal cells; ADCC, antibody-dependent cellular cytotoxicity.

Response rates to γδT cell-based immunotherapy, either as adoptive transfer or as stimulating drugs, are not satisfactory (10% of objective responses); however, about 39% of patients achieved disease stabilization, indicating a clinical benefit and suggesting the possibility of improving the efficiency of such therapeutic tool (56, 58, 80). Advantages of this type of anti-cancer therapy would be the safety of drugs and substances known to stimulate γδT cells, beside their efficiency in γδT-cell stimulation. Drawbacks are mainly represented by immunoevasion. This can be counteracted (Figure 1) by including in the therapeutic protocols non-specific stimulators as TLR agonists (imiquimod or resiquimod) or the BCG vaccine (81). Recently approved clinical trials include mAbs blocking PD-1, PDL-1, and CTLA-4 (58, 70, 71) aimed to inhibit negative signals. Cancer-specific TCR gene transfer has been proposed in the last years to gain efficiency and specificity in the anti-cancer response; αβTCR engineered γδT cells have been shown to exert anti-tumor activity *in vitro* and may be considered as an alternative strategy for adoptive T-cell transfer (82, 83).

The recent identification of BTN3A1 as an essential molecule in P-Ag presentation to γδT cells opens new possible ways of interventions: both stimulating and inhibiting mAbs directed to the B30.2 basic domain of the molecule have been described (39–41, 84). These antibodies might be used differently to induce or regulate γδ T-cell response to P-Ag, representing an additional tool in the design of immunotherapeutic protocols.

In addition, we propose the use of N-BPs not only as γδT-cell stimulating agents but as immunomodulating drugs (79). Finally, the development of ADAMs specific and non-toxic inhibitors would contribute to the improvement of NKG2D-mediated recognition of stress-induced molecules at the surface of tumor cells. Thus, such combined therapeutic protocols, including stimulating molecules, mAbs, and inhibitory substances acting on enzymes, which favor tumor immunoevasion, may represent the new frontier of anti-cancer immunotherapy.

## Conflict of Interest Statement

The authors declare that the research was conducted in the absence of any commercial or financial relationships that could be construed as a potential conflict of interest.
